# Analysis of Clinical Characteristics, Treatment, and Prognostic Factors of 106 Breast Cancer Patients With Solitary Pulmonary Nodules

**DOI:** 10.3389/fsurg.2022.843913

**Published:** 2022-02-15

**Authors:** Lihong He, Xiaorui Wang, Xiaodong Liu, Yongsheng Jia, Weipeng Zhao, Xiaochen Jia, Yuehong Zhu, Wenjing Meng, Zhongsheng Tong

**Affiliations:** Key Laboratory of Cancer Prevention and Therapy, Department of Breast Cancer, National Research Center for Cancer, Tianjin Medical University Cancer Institute and Hospital, Tianjin, China

**Keywords:** breast cancer, primary lung cancer, metastatic pulmonary breast cancer, treatment, prognosis

## Abstract

**Objective:**

The clinical features of solitary pulmonary nodules (SPN) in breast cancer patients were retrospectively analyzed, and the clinical features of primary lung cancer (PLC) and metastatic pulmonary breast cancer (MBC) in breast cancer patients were compared, and the treatment plan, curative effect and influencing factors were analyzed.

**Methods:**

The clinical data of 106 patients of SPN combined with breast cancer surgery in our hospital from January 2015 to June 2020 were analyzed. There were 65 patients of PLC and 41 patients of MBC. Record the characteristics of the primary breast cancer lesion in our patient, the interval between the initial diagnosis of breast cancer and the appearance of SPN, the previous treatment history of our patient, and the characteristics and surgical method of SPN. The survival status of all patients during the follow-up period was recorded.

**Results:**

The onset age, interval, maximum nodule diameter, ER expression positive rate and radiotherapy history ratio of PLC patients were higher than those of MBC patients, and the lymph node positive rate and triple negative rate were lower than those of MBC patients (*P* < 0.05). Median survival was 51 months in patients with PLC and 37 months in patients with MBC. The 1, 3, and 5 year overall survival rates in patients with PLC were higher than those in patients with MBC (*P* < 0.05). Vascular tumor thrombus, SPN type and chemotherapy were all independent factors affecting the prognosis of patients with breast cancer combined with SPN (*P* < 0.05).

**Conclusion:**

PLC patients and MBC patients have significant differences in pathological characteristics, like the onset age, interval, maximum nodule diameter, ER expression positive rate, radiotherapy history ratio, the lymph node positive rate, and triple negative rate. Septum, vascular tumor thrombus, SPN type, and chemotherapy are all independent factors that affect the curative effect of breast cancer patients with SPN. Based on the nature of SPN, it can provide reference for clinicians to decide the treatment plan, improve patients' quality of life and prolong their survival time.

## Introduction

Breast cancer is one of the most common malignant tumors in women, and its incidence is extremely high (1). With the application of radiotherapy, chemotherapy, and targeted therapy, the survival time of breast cancer patients is prolonged, but the risk of secondary primary cancer is increasing gradually. Studies have shown that the primary malignant tumors secondary to breast cancer mainly include endometrial cancer, blood tumor, lung cancer, and so on (2). It is the second most common primary cancer patients with breast cancer, and the lung is also the common metastatic site of breast cancer (3, 4). When an isolated pulmonary nodule (SPN) is found in patients with breast cancer, it is easy to diagnose metastatic pulmonary breast cancer (MBC) clinically, and the treatment direction will be biased toward breast cancer, leading to the change of treatment choice (5). The clinical diagnosis and treatment of MBC need to be differentiated, but the epidemiological and clinicopathological features of secondary lung cancer after breast cancer have not been fully revealed, and multi-center research lacks the support of big data. Therefore, when finding SPN in breast cancer patients, the first clinical problem to be solved to accurately diagnose the nature of SPN and distinguish between primary lung cancer (PLC) and MBC, which is of great significance to guide breast cancer patients and SPN patients to choose the best individualized treatment scheme (6, 7). In this study, we retrospectively analyzed the clinical characteristics of breast cancer patients with SPN, compared the clinical characteristics of PLC and MBC, and analyzed the efficacy of patients and the related factors affecting the prognosis of breast cancer patients with SPN, in order to provide clinical basis for the treatment and prognosis of breast cancer patients with SPN.

## Data and Methods

### General Information

The clinical data of 106 patients of SPN combined with breast cancer diagnosed and treated in our hospital from January 2015 to June 2020 were analyzed. All patients chose the treatment plan according to their specific condition, and they met the patient inclusion criteria.

#### Inclusion Criteria

SPN was found during postoperative follow-up of breast cancer, and confirmed as PLC or MBC; By biopsy or post-operative pathological examination; Breast cancer was the first primary cancer; Clinical data of diagnostic immunohistochemistry; and postoperative treatment of breast cancer were complete.

#### Exclusion Criteria

Benign pulmonary nodules diagnosed by pathological examination; Patients with history of metastases of other malignant tumors or other sites; Follow-up universal periodic review failed; and follow-up data was lost.

All the patients were female, and 65 patients with PLC and 41 patients with MBC were diagnosed pathologically from SPN. Among the 65 PLC patients, there were 29 patients with lung squamous cell carcinoma, 22 patients with lung adenocarcinoma, 11 patients with small cell lung cancer, and 3 patients with large cell lung cancer.

### Research Methods

The characteristics of primary breast cancer lesions in our patient were recorded, including pathological type, histological grade, lymph node status, expression of estrogen receptor (ER), progesterone receptor (PR), human epidermal growth factor receptor 2 (HER-2), and vascular tumor thrombi. HER-2 was determined to be negative based on 0 or + by immunohistochemistry and positive based on +++ by fluorescence *in situ* hybridization. ER or PR positivity is defined as the proportion of cells positive for immunohistochemical staining reaching more than 1%. The triple negativity was negative for ER, PR, and HER-2. Record the interval between the initial diagnosis of breast cancer and the appearance of SPN, the previous treatment history of the patient, and the characteristics and surgical method of SPN. Patients were followed up to June 2021, and their post-treatment survival (from surgery to time of death or follow-up deadline) was recorded.

### Statistical Methods

SPSS22.0 software was used for processing. The measurement data of experimental data were expressed as mean standard deviation ($\bar{x}\pm$ s), and the enumeration data were expressed as (%). *t*-test was used for pairwise comparison of measurement data between groups, and χ^2^ test was used for enumeration data. Kaplan-Meier method was used to draw the survival curve. Multivariate Logisitic regression model was used to analyze the related factors affecting the postoperative efficacy of patients with breast cancer combined with SPN. The test level was α = 0.05, and *P* < 0.05 indicated that the difference was statistically significant.

## Results

### Comparison of Clinical Characteristics Between Patients With PLC and MBC Disease

The differences in age at onset, interval, maximum nodule diameter, lymph node status, ER expression, triple negative, and radiotherapy history between PLC patients and MBC patients were statistically significant (*P* < 0.05), while the differences were not statistically significant in pathological type, histological grade, PR expression, HER-2 expression, chemotherapy history, and endocrine treatment history (*P* > 0.05) as shown in Table 1.

**Table 1:** Comparison of clinical characteristics between patients with PLC and MBC disease (*n*, $\bar{x}\pm$ s).

**Clinical pathological features**	**PLC (*n* = 65)**	**MBC (*n* = 41)**	***t/χ^2^* value**	***P-*value**
Onset age (years)	55.42 ± 11.08	43.72 ± 9.85	5.523	0.027
Interval (years)	5.47 ± 1.53	2.86 ± 1.07	6.542	0.008
Maximum diameter of nodule (mm)	22.39 ± 8.94 patients with	16.24 ± 10.53	3.218	0.042
Pathological type of breast cancer			0.239	0.624
Invasive ductal carcinoma	48 (73.85%)	32 (78.05%)		
Invasive lobular carcinoma	17 (26.15%)	9 (21.95%)		
Histological grading			0.025	0.985
Level 1	12 (18.46%)	8 (19.51%)		
Level 2	31 (47.69%)	19 (46.34%)		
Level 3	22 (33.85%)	14 (34.15%)		
Lymph node status			6.154	0.013
Positive	30 (46.15%)	29 (70.73%)		
Negative	35 (53.85%)	12 (29.27%)		
ER expression			4.391	0.036
Positive	42 (64.62%)	18 (43.90%)		
Negative	23 (35.38%)	23 (56.10%)		
PR expression			0.331	0.566
Positive	37 (56.92%)	21 (51.22%)		
Negative	28 (43.08%)	20 (48.78%)		
HER-2 expression			0.095	0.758
Positive	25 (38.46%)	17 (41.46%)		
Negative	40 (61.54%)	24 (58.54%)		
Triple negative			4.352	0.037
Yes	12 (18.46%)	15 (36.59%)		
No	53 (81.54%)	26 (63.41%)		
History of radiotherapy			5.221	0.029
Yes	37 (56.92%)	16 (39.02%)		
No	28 (43.08%)	25 (60.98%)		
Chemotherapy history			0.351	0.554
Yes	58 (89.23%)	38 (92.68%)		
No	7 (10.77%)	3 (7.32%)		
History of endocrine therapy			2.107	0.146
Yes	44 (67.69%)	22 (53.66%)		
No	21 (32.31%)	19 (46.34%)		

### Treatment of Patients With PLC and MBC Disease

Among 65 patients with primary liver cancer, 23 patients underwent radical lobectomy plus chemotherapy plus radiotherapy, 39 patients underwent wedge resection plus chemotherapy, and 3 patients underwent radiotherapy plus chemotherapy. Among 41 patients with MBC, 13 patients received wedge resection plus endocrine therapy, 21 patients received chemotherapy plus radiotherapy plus endocrine therapy, and seven patients received radiotherapy and chemotherapy.

### Prognosis of Patients With PLC and MBC Disease

**Figure 1:**
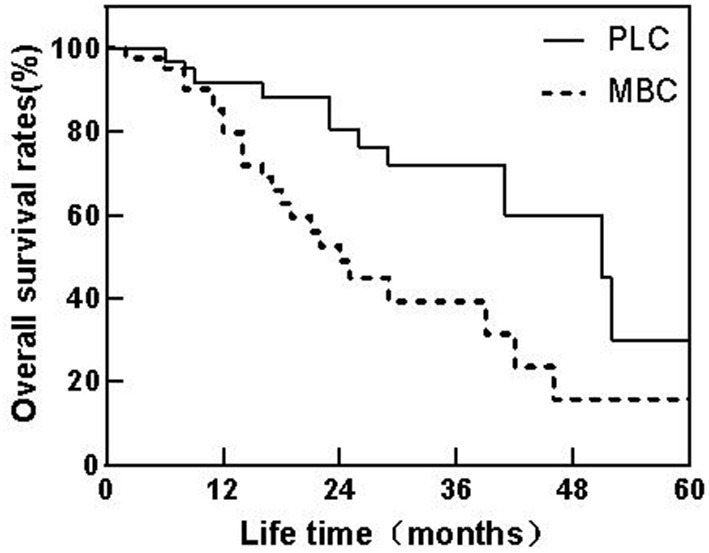
Patient survival curves of PLC and MBC.

The median survival time of patients with PLC was 51 months, and the overall survival rates of 1, 3, and 5 years were 90.77, 72.31, and 30.77%, respectively. The median survival time of patients with MBC was 37 months, and the overall survival rates of 1, 3, and 5 years were 82.93, 41.46, and 19.51%, respectively. The median survival time of PLC patients is longer than that of MBC patients, and the Log-rank test is *P* = 0.034, the difference is statistically significant. The 1, 3, and 5 years overall survival rates of patients with PLC were higher than those of patients with MBC, and the differences were statistically significant (*P* < 0.05) as shown in Table 2, Figure 1.

**Table 2:** Comparison of overall survival rates between patients with PLC and MBC disease (*n*, %).

**Group**	**Overall survival rate**		
	**1 year**	**3 years**	**5 years**
PLC (n = 65)	59 (90.77%)	47 (72.31%)	20 (30.77%)
MBC (n = 41)	34 (82.93%)	17 (41.46%)	8 (19.51%)
*χ^2^-*value	3.437	5.998	5.512
*P-*value	0.041	0.012	0.016

### Univariate Analysis of Prognosis of Patients With Breast Cancer Combined With SPN

Univariate analysis showed that the prognosis of breast cancer patients with SPN was related to the interval time, the presence or absence of vascular tumor thrombus, the maximum diameter of nodules, the type of SPN and chemotherapy (*P* < 0.05) as shown in Table 3.

**Table 3:** Univariate analysis of prognosis of patients with breast cancer combined with SPN (*n*, %).

**Clinical pathological features**	**Survival (*n* = 28)**	**Death (*n* = 78)**	***χ^2^-*value**	***P-*value**
Onset age (years)			0.394	0.531
≥50	17 (60.71%)	42 (53.85%)		
<50	11 (39.29%)	36 (46.15%)		
Interval time (years)			10.118	0.001
≥3	22 (78.57%)	34 (43.59%)		
<3	6 (21.43%)	44 (56.41%)		
Vascular cancer thrombi			13.562	<0.001
Yes	7 (25.00%)	51 (65.38%)		
No	21 (75.00%)	27 (34.62%)		
Pathological type			1.192	0.275
Invasive ductal carcinoma	19 (67.86%)	61 (78.21%)		
Invasive lobular carcinoma	9 (32.14%)	17 (21.79%)		
Histological grading			1.088	0.581
Level 1	7 (25.00%)	13 (16.67%)		
Level 2	13 (46.43%)	37 (47.44%)		
Level 3	8 (28.57%)	28 (35.89%)		
Lymph node status			0.034	0.853
Positive	16 (57.14%)	43 (55.13%)		
Negative	12 (42.86%)	35 (44.87%)		
Maximum diameter of nodule (mm)			5.782	0.016
≥20	22 (78.57%)	41 (52.56%)		
<20	6 (21.43%)	37 (47.44%)		
Type of SPN			4.639	0.024
PLC	20 (71.43%)	45 (57.69%)		
MBC	8 (28.57%)	33 (42.31%)		
Surgical approach			0.535	0.464
Lobectomy	10 (35.71%)	13 (16.67%)		
Wedge resection/tumor resection	18 (64.29%)	34 (43.59%)		
Chemotherapy			5.319	0.021
Yes	28 (100.00%)	65 (83.33%)		
No	0 (0.00%)	13 (16.67%)		
Endocrine therapy			0.063	0.802
Yes	6 (21.43%)	15 (19.23%)		
No	22 (78.57%)	63 (80.77%)		

### Analysis of Multiple Factors Affecting the Prognosis of Patients With Breast Cancer Combined With SPN

Multivariate analysis showed that interval, vascular tumor thrombus, the type of SPN, and chemotherapy were all independent factors affecting the prognosis of patients with breast cancer combined with SPN (*P* < 0.05) as shown in Tables 4, 5.

**Table 4:** Assignment for multivariate analysis of factors.

**Factors**	**Variables**	**Assignment**
Interval time	X1	≥3 years = 0, <3 years = 1
Vascular tumor thrombus	X2	Yes = 1, No = 0
Maximum diameter of nodules	X3	≥20 mm = 0, <20 mm = 1
Type of SPN	X4	PLC = 0, MBC = 1
Chemotherapy	X5	Yes = 1, No = 0

**Table 5:** Multi-factor analysis of prognosis of patients with breast cancer combined with SPN.

**Factor**	***B***	***SE***	***Walds***	***P***	***OR***	***95% CI***
Interval time	2.381	1.053	5.113	0.031	10.816	1.373–15.190
Vascular tumor thrombus	2.195	0.956	5.272	0.024	8.980	1.379–9.484
Maximum diameter of nodules	1.086	0.734	2.189	0.298	2.962	0.702–12.486
Type of SPN	2.534	0.862	8.642	0.016	12.604	2.327–18.273
Chemotherapy	2.254	0.864	6.806	0.018	9.526	1.752–11.803

## Discussion

Lung metastasis is a common site of postoperative recurrence and metastasis of breast cancer. The differentiation of SPN in breast cancer patients has always been a difficult problem (8). Clinically, when looking for SPN in patients with a breast cancer history, the first thing to consider is usually the occurrence of lung metastasis from breast cancer. However, for SPN in the lung, breast cancer combined is mostly combined with PLC, and MBC or benign lung diseases are the second most important place (9). Studies have shown that compared with the normal population, breast cancer patients are more likely to be accompanied by PLC, which may be related to the common genetic factors and hormone genes of the two diseases, and has a certain correlation with the survival time of breast cancer patients (10). With the development and popularization of modern video-assisted thoracoscopy technology, the clinical diagnosis rate of SPN is increasing. Obtaining pathological diagnosis is the gold standard for clinical diagnosis. Therefore, for SPN whose nature cannot be clearly defined, it is feasible to first remove the focus and make a definite diagnosis. According to SPN, the next treatment plan includes anti-inflammation, endocrine therapy, chemotherapy, and targeted therapy (11, 12). Accurate identification of PLC or MBC in time, and determination of the next treatment plan according to the nature of SPN, is an important basis for rational clinical treatment (13).

In this study, the clinical data of patients with breast cancer complicated with PLC and MBC were retrospectively analyzed. The results showed that there were significant differences between PLC patients and MBC patients in onset age, interval, maximum nodule diameter, lymph node status, ER expression, triple negative, and radiotherapy history and so on. Compared with MBC patients, PLC patients have older onset age, longer interval and larger nodule diameter, which may be related to the preference of PLC for elderly patients, as well as the fact that some advanced MBC patients have more tumors are prone to lung metastasis (14, 15). Patients with negative lymph nodes, positive ER expression, less triple-negative, and more radiotherapy history are more likely to be diagnosed with PLC, which may be related to the longer survival time of these patients, and chemotherapy significantly reduces the probability of local recurrence of breast cancer (16, 17). Radiotherapy plays an important role in reducing local recurrence of breast cancer and increasing survival rates. Studies have shown that chemotherapy can lead to an increased incidence of Hodgkin's lymphoma complicated with lung cancer. In addition, estrogen is closely related to the occurrence of lung cancer, and many lung cancer cells are also accompanied by over-expression of ER (especially ERβ) (18, 19).

Excision of the lesion can clearly diagnose the nature of SPN, so as to determine the next treatment plan in clinic, including radiotherapy, chemotherapy, and endocrine therapy (20). Among 65 PLC patients, 23 patients underwent radical lobectomy + chemotherapy + radiotherapy, 39 patients underwent wedge resection + chemotherapy, and three patients underwent radiotherapy + chemotherapy. Among 41 MBC patients, 13 underwent wedge resection plus endocrine therapy, 21 underwent chemotherapy plus radiotherapy plus endocrine therapy, and seven underwent radiotherapy plus chemotherapy. The 5-year overall survival rate of patients with PLC was significantly higher than that of MBC patients. For breast cancer patients combined with PLC, surgical resection of lung diseases is an important means of treatment (21). For MBC patients, general palliative treatment is mainly used, and chemotherapy and endocrine therapy can effectively improve the survival rate (22). If the surgical treatment can be tolerated, whether surgical resection of solitary pulmonary metastases is meaningful to improve the quality of life and prolonging the survival time of MBC patients remains to be verified by prospective research results. The survival time of PLC in breast cancer is mainly determined by lung cancer. After radical resection, the clinical prognosis is good and the survival rate is high within 5 years. Once MBC appears, it is divided into the fourth stage. Even after comprehensive treatment, the prognosis is poor and the overall survival rate is significantly lower than that of breast cancer combined with PLC patients (23, 24).

The results of this study show that the curative effect of breast cancer patients with SPN was related to the interval time, the presence or absence of vascular tumor thrombus, the maximum diameter of nodules, the type of SPN, chemotherapy, and other factors. Further multivariate analysis showed that interval time, vascular tumor thrombus, SPN type, and chemotherapy were independent factors that affected the curative effect of breast cancer patients with SPN. The longer the interval between the diagnosis of breast cancer and the discovery of SPN, the PLC nature of SPN in breast cancer patients with SPN show the characteristics of better prognosis and longer survival time (25). The absence of vascular tumor thrombi and the longer survival time of patients undergoing chemotherapy may be related to the fact that the tumor load of patients is low, and chemotherapy is conducive to the removal of micro metastases throughout the body.

To sum up, compared with MBC patients, there are significant differences in the onset age, interval, maximum diameter of nodules, lymph node status, ER expression, triple negative, and radiation history among PLC patients. Septum, vascular tumor thrombus, SPN type and chemotherapy are all independent factors that affect the curative effect of breast cancer patients with SPN. Based on the nature of SPN, it can provide reference for clinicians to decide the treatment plan, improve patients' quality of life and prolong their survival time.

## Data Availability Statement

The original contributions presented in the study are included in the article/supplementary material, further inquiries can be directed to the corresponding author.

## Ethics Statement

The studies involving human participants were reviewed and approved by the Medical Ethics Committee of Tianjin Medical University Cancer Institute and Hospital. The patients/participants provided their written informed consent to participate in this study.

## Author Contributions

LH and XW were mainly responsible for the design of the study and the writing of the manuscript. XL and YJ were mainly responsible for the collection of case data. WZ and XJ were responsible for the data recording. YZ and WM were responsible for the data statistical analysis. ZT was the instructor of the whole study. All authors contributed to the article and approved the submitted version.

## Funding

This study was supported by Key Task Project of Tianjin Health and Family Planning Commission (16KG128), Anticancer Key Technologies R&D Program of Tianjin (12ZCDZSY16200), Natural Science Foundation of Tianjin (18JCYBJC91600), amd Scientific research program of Tianjin Education Commission (2020KJ139).

## Conflict of Interest

The authors declare that the research was conducted in the absence of any commercial or financial relationships that could be construed as a potential conflict of interest.

## Publisher's Note

All claims expressed in this article are solely those of the authors and do not necessarily represent those of their affiliated organizations, or those of the publisher, the editors and the reviewers. Any product that may be evaluated in this article, or claim that may be made by its manufacturer, is not guaranteed or endorsed by the publisher.

## References

[1] Tsang JYS Tse GM. Molecular Classification of Breast Cancer. Adv Anat Pathol. (2020) 27:27–35. 10.1097/PAP.0000000000000232 31045583

[2] Cai WL Greer CB Chen JF Arnal-Estapé A Cao J Yan Q. Specific chromatin landscapes and transcription factors couple breast cancer subtype with metastatic relapse to lung or brain. BMC Med Genomics. (2020) 13:33. 10.1186/s12920-020-0695-0 32143622 PMC7060551

[3] Jin L Han B Siegel E Cui Y Giuliano A Cui X. Breast cancer lung metastasis: Molecular biology and therapeutic implications. Cancer Biol Ther. (2018) 19:858–68. 10.1080/15384047.2018.1456599 29580128 PMC6300341

[4] Medeiros B Allan AL. Molecular mechanisms of breast cancer metastasis to the lung: clinical and experimental perspectives. Int J Mol Sci. (2019) 20:2272. 10.3390/ijms20092272 31071959 PMC6540248

[5] Peart O. Metastatic breast cancer. Radiol Technol. (2017) 88:519–39. 28500107

[6] Liu C Li H Xu K Song S He Y Cai X. Multiple primary lung cancer versus intrapulmonary metastatic cancer: a case of multiple pulmonary nodules. Thorac Cancer. (2019) 10:352–8. 10.1111/1759-7714.12918 30548923 PMC6360236

[7] Yousefi M Nosrati R Salmaninejad A Dehghani S Shahryari A Saberi A. Organ-specific metastasis of breast cancer: molecular and cellular mechanisms underlying lung metastasis. Cell Oncol. (2018) 41:123–40. 10.1007/s13402-018-0376-6 29568985 PMC12995240

[8] Li K Xu C Du Y Junaid M Kaushik AC Wei DQ. Comprehensive epigenetic analyses reveal master regulators driving lung metastasis of breast cancer. J Cell Mol Med. (2019) 23:5415–31. 10.1111/jcmm.14424 31215771 PMC6653217

[9] Macherey S Mallmann P Malter W Doerr F Heldwein M Wahlers T. Lung metastasectomy for pulmonary metastatic breast carcinoma. Geburtshilfe Frauenheilkd. (2017) 77:645–50. 10.1055/s-0043-108252 28769127 PMC5489404

[10] Neves M Fumagalli A van den Bor J Marin P Smit MJ Mayor F. The role of ACKR3 in 8. Mol Pharmacol. (2019) 96:819–25. 10.1124/mol.118.115279 30745320

[11] Xu H Pu XH Yu TF Shi HB Wu YL Xu YM. Incidence and natural course of CT-detected pulmonary ground-glass nodules in Chinese women with breast cancer: a retrospective, single-center, long-term follow-up study in 4682 consecutive patients. Acta Radiol. (2020) 61:175–83. 10.1177/0284185119856259 31216178

[12] Hammer MM Mortani Barbosa EJ Jr. Predictive factors for malignancy in incidental pulmonary nodules detected in breast cancer patients at baseline CT. Eur Radiol. (2017) 27:2802–9. 10.1007/s00330-016-4627-5 27798753

[13] Song Z Ye T Ma L Xiang J Chen H. Surgical outcomes of isolated malignant pulmonary nodules in patients with a history of breast cancer. Ann Surg Oncol. (2017) 24:3748–53. 10.1245/s10434-017-6067-0 28849376

[14] Lin S Mo H Li Y Guan X Chen Y Wang Z. Clinicopathological characteristics and survival outcomes in patients with synchronous lung metastases upon initial metastatic breast cancer diagnosis in Han population. BMC Cancer. (2021) 21:1330. 10.1186/s12885-021-09038-2 34906122 PMC8670055

[15] He KW Wei W Liu ZY Song X Zhuo PY Ma QH. [Clinicopathological features of second primary lung cancer and pulmonary metastasisin patients with breast cancer]. Zhonghua Zhong Liu Za Zhi. (2018) 40:201–5. 10.3760/cma.j.issn.0253-3766.2018.03.008 29575839

[16] Kwapisz D. Oligometastatic breast cancer. Breast Cancer. (2019) 26:138–46. 10.1007/s12282-018-0921-1 30324552

[17] Liu B Xia H. Progress in Surgery for Pulmonary Metastases. Zhongguo Fei Ai Za Zhi. (2019) 22:574–8. 10.3779/j.issn.1009-3419.2019.09.04 31526461 PMC6754572

[18] Xie L Lin C Zhang H Bao X. Second malignancy in young early-stage breast cancer patients with modern radiotherapy: a long-term population-based study (A STROBE-compliant study). Medicine. (2018) 97:593. 10.1097/MD.0000000000010593 29703057 PMC5944535

[19] Meng W Liao Y Chen J Wang Y Meng Y Li K. Upregulation of estrogen receptor beta protein but not mRNA predicts poor prognosis and may be associated with enhanced translation in non-small cell lung cancer: a systematic review and meta-analysis. J Thorac Dis. (2021) 13:4281–300. 10.21037/jtd-21-658 34422356 PMC8339768

[20] Latorzeff I Bourgier C Pinel B Hennequin C Jimenez G Chapet O. Traitement de la maladie primitive (cancers du sein, du poumon non à petites cellules et de la prostate), par irradiation, au stade d'emblée métastatique [Treatment of primary disease (breast, non-small cell lung and prostate cancers) with irradiation in case of de novo metastatic cancer]. Cancer Radiother. (2019) 23:486–95. 10.1016/j.canrad.2019.08.004 31501025

[21] Cruickshank A Stieler G Ameer F. Evaluation of the solitary pulmonary nodule. Intern Med J. (2019) 49:306–15. 10.1111/imj.14219 30897667

[22] Xiao W Zheng S Liu P Zou Y Xie X Yu P. Risk factors and survival outcomes in patients with breast cancer and lung metastasis: a population-based study. Cancer Med. (2018) 7:922–30. 10.1002/cam4.1370 29473333 PMC5852337

[23] Wang JC Li GY Wang B Han SX Sun X Jiang YN. Metformin inhibits metastatic breast cancer progression and improves chemosensitivity by inducing vessel normalization via PDGF-B downregulation. J Exp Clin Cancer Res. (2019) 38:235. 10.1186/s13046-019-1211-2 31164151 PMC6549289

[24] Urooj T Wasim B Mushtaq S Shah SNN Shah M. Cancer cell-derived secretory factors in breast cancer-associated lung metastasis: their mechanism and future prospects. Curr Cancer Drug Targets. (2020) 20:168–86. 10.2174/1568009620666191220151856 31858911 PMC7516334

[25] Chen MT Sun HF Zhao Y Fu WY Yang LP Gao SP. Comparison of patterns and prognosis among distant metastatic breast cancer patients by age groups: a SEER population-based analysis. Sci Rep. (2017) 7:9254. 10.1038/s41598-017-10166-8 28835702 PMC5569011

